# A microgel-stabilized, light-controlled artificial energy supply module for efficient biosynthesis

**DOI:** 10.1093/rb/rbaf106

**Published:** 2025-11-08

**Authors:** Shaoyang Kang, Sheng Ding, Donghao Lyu, Rui Gao, Sirui Peng, Jing Liu, Chuangnian Zhang, Zujian Feng, Pingsheng Huang, Deling Kong, Weiwei Wang

**Affiliations:** State Key Laboratory of Advanced Medical Materials and Devices, Institute of Biomedical Engineering, Chinese Academy of Medical Sciences and Peking Union Medical College, Tianjin 300192, China; College of Life Sciences, Key Laboratory of Bioactive Materials (Ministry of Education), State Key Laboratory of Medicinal Chemical Biology, Nankai University, Tianjin 300071, China; State Key Laboratory of Advanced Medical Materials and Devices, Institute of Biomedical Engineering, Chinese Academy of Medical Sciences and Peking Union Medical College, Tianjin 300192, China; State Key Laboratory of Advanced Medical Materials and Devices, Institute of Biomedical Engineering, Chinese Academy of Medical Sciences and Peking Union Medical College, Tianjin 300192, China; State Key Laboratory of Advanced Medical Materials and Devices, Institute of Biomedical Engineering, Chinese Academy of Medical Sciences and Peking Union Medical College, Tianjin 300192, China; State Key Laboratory of Advanced Medical Materials and Devices, Institute of Biomedical Engineering, Chinese Academy of Medical Sciences and Peking Union Medical College, Tianjin 300192, China; State Key Laboratory of Advanced Medical Materials and Devices, Institute of Biomedical Engineering, Chinese Academy of Medical Sciences and Peking Union Medical College, Tianjin 300192, China; College of Life Sciences, Key Laboratory of Bioactive Materials (Ministry of Education), State Key Laboratory of Medicinal Chemical Biology, Nankai University, Tianjin 300071, China; State Key Laboratory of Advanced Medical Materials and Devices, Institute of Biomedical Engineering, Chinese Academy of Medical Sciences and Peking Union Medical College, Tianjin 300192, China; College of Life Sciences, Key Laboratory of Bioactive Materials (Ministry of Education), State Key Laboratory of Medicinal Chemical Biology, Nankai University, Tianjin 300071, China; College of Life Sciences, Key Laboratory of Bioactive Materials (Ministry of Education), State Key Laboratory of Medicinal Chemical Biology, Nankai University, Tianjin 300071, China

**Keywords:** artificial organelle, microgel, light-activation, ATP production

## Abstract

Artificial energy supply modules that can produce adenosine triphosphate (ATP) through natural or synthetic structures are crucial for supporting artificial cells with therapeutic purposes. However, their advanced biomedical application is hindered by poor stability, short lifespan and low output efficiency. In this study, an artificial light-controlled energetic module with long-term activity, termed thylakoid-loaded microgel (TM), is created by encapsulating spinach-derived thylakoid into alginate/gelatin microgels. The TM effectively retains the photosynthetic light reactions of thylakoids, including the electron transfer capacity of photosystem II and ATP production, and releases the produced ATP to external environment. As a proof of concept, the TM successfully drives the luciferin/luciferase reaction both within and outside the microgel compartment. In addition, the encapsulated thylakoids exhibit a significantly prolonged activity, with the high photosystem II activity and ATP production lasting for at least 96 h. The long-term activity is attributed to the oxidation shielding efficacy, protein and pigment degradation inhibition and membrane structural stabilization. This study presents a strategy for developing artificial energy supply modules with efficient energy output and long-term activity, holding great promise in artificial cell construction and biosynthesis.

## Graphical abstract

**Figure:**
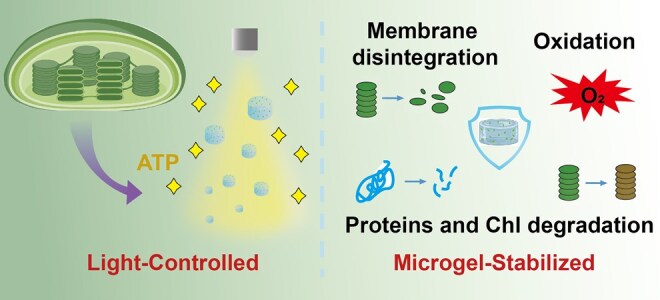

## Introduction

Micro-scale artificial living systems, including man-made cells and organelles, are assembled from synthetic or natural components to partly mimic the functions of natural cells or organelles [1–4]. These systems have gained increasing attention for both studying the fundamental processes in natural cells and treating diseases in recent years [5–9]. Similar to their natural counterparts, the functionality of artificial cells or organelles depends on energy, typically in the form of adenosine triphosphate (ATP) generated by energy supply modules, to sustain enzymatic processes occurring within them. Based on the origins of energy, the energy supply modules can be categorized into two types: chemical- and photo-driven modules [10]. The chemical-driven modules function on the basis of oxidative phosphorylation, which converts the chemical energy stored in substrates, such as glucose, into ATP. The photo-driven modules are constructed on the light reactions of photosynthesis, which require photoconverters, such as photosystem II (PSII) and bacteriorhodopsin, to capture light energy for ATP production [11, 12]. Both types of modules rely on an intact electron transfer chain (ETC) to generate a proton gradient that drives ATP synthesis. The ETC within the modules can be fully synthetic and assembled via a bottom-up method, wherein essential enzymes, protein complexes and cofactors are artificially combined and incorporated into a designed compartment [13, 14]. Another strategy to obtain ETC is to integrate natural components containing ETC, such as mitochondria and thylakoids, into biomaterials to construct a semi-synthetic module [9, 15–17]. Compared with chemical-driven modules, the photo-driven modules derived from plants are more environmentally friendly without the demand for energy-stored substrates [10]. In addition, the construction of semi-synthetic modules is simpler and more cost-effective than the fully synthetic modules, which require synthesis or isolation of various enzymes and proper topological orientations when inserting them into compartments to form a functional system [18, 19]. Therefore, the artificial living systems based on semi-synthetic or photo-driven modules have attracted great interest in a wide range of applications, including carbon fixation and metabolism improvement [20–24].

However, there are several issues limiting their advanced applications. Firstly, the energy supply is limited within the compartments and lacks effective outputs to external environments. Currently, most compartments are constructed by liposomes and coacervates [7]. The impermeability of liposome-based compartments to polar and charged molecules limits the transportation of ATP to other systems. Passive diffusion of ATP or other products contributes to a relatively low utilization efficiency. Secondly, the long-term activity is constrained by the rapid inactivation of bioactive components. For instance, isolated mitochondria will lose the function within several hours after extraction under the deterrence of oxidative stress and osmotic damage [25–27]. For coacervate-based compartments, thylakoids encapsulated in coacervate-based compartments also did not exhibit a significantly prolonged lifetime compared to naked ones. These limitations have largely restricted the advancement and translation of artificial living systems. Therefore, developing long-lived energy supply modules with efficient energy output capacity is crucial.

The involvement of hydrogels has shown great potentials to alleviate these limitations. In contrast to liposomes, hydrogels can encapsulate large molecules such as enzymes, while allowing for the exchange of small molecules [28]. Consequently, microgels (micro-scale hydrogels) have been utilized as the compartments of artificial living systems with more pronounced interactivity with external environments [29]. Furthermore, hydrogel encapsulation creates a stable but partly isolated environment, which has shown the efficacy of protecting bioactive components from detrimental factors in mitochondrial transplantation [30, 31]. Westensee *et al.* have reported an artificial energy module based on mitochondria encapsulated in gelatin methacryloyl microgels with a long lifetime of at least 24 h [15]. While the liposome-based compartments also extend the activity retention time, they cannot ensure effective interaction with external environments [17]. Thus, microgels are expected to be promising candidates for constructing artificial energy modules.

**Figure 1:**
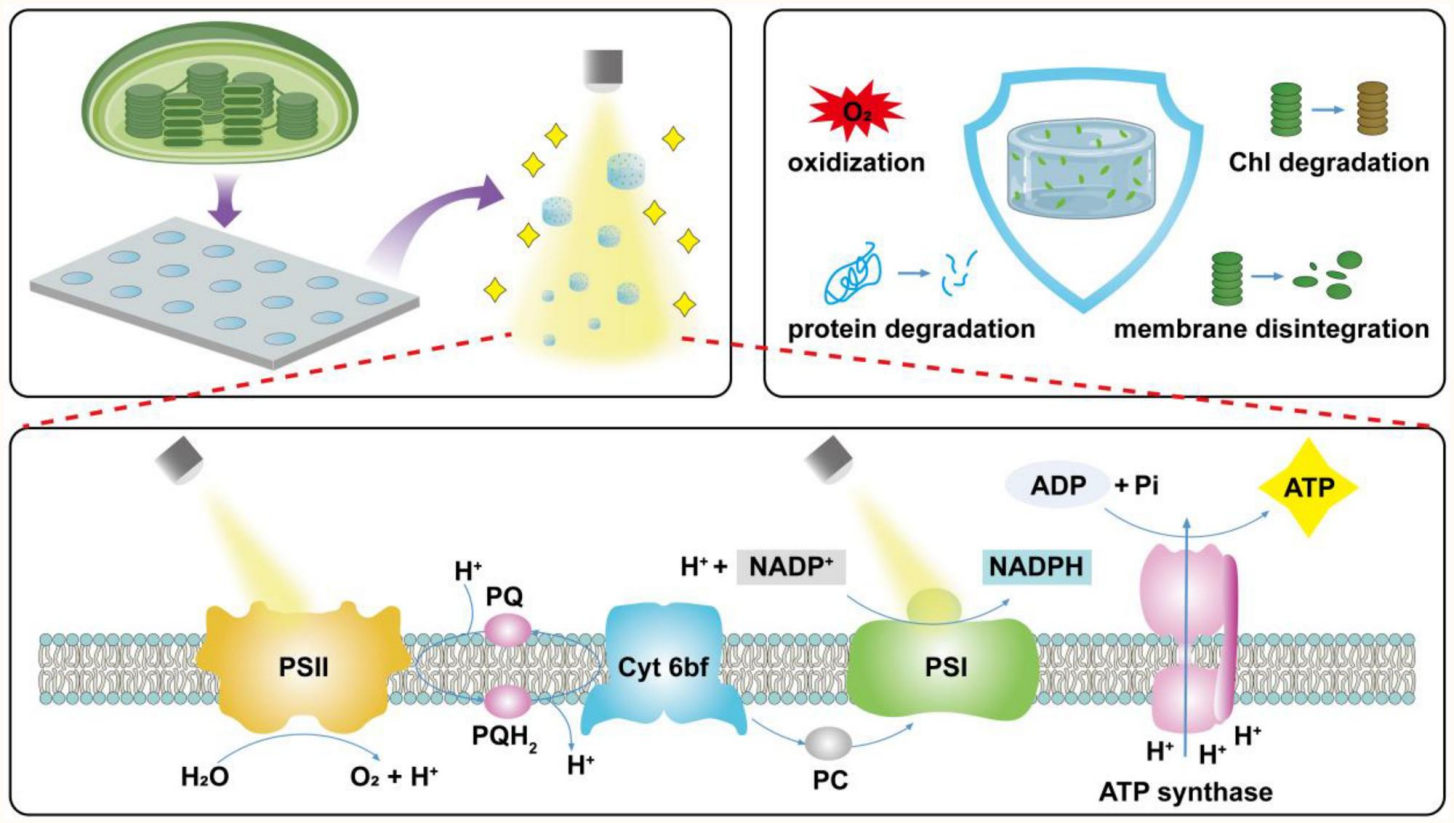
Illustration of the artificial light-controlled energy supply module with long-term activity based on microgels. Briefly, thylakoids were isolated from fresh spinach leaves and then encapsulated into Alg/Gel microgels through a micromold method. The microgels inherited the ability of producing ATP under light irritation and were capable of releasing ATP to the outside environment and prolonging the activity retention time through oxidation shielding effect, inhibition of protein and pigment degradation, and structure stabilization.

Herein, we report a semi-synthetic, light-controlled energy supply module by encapsulating thylakoids into alginate/gelatin (Alg/Gel) microgels, enabling a long-term activity and effective energy output to external environments (Figure 1). The modules fully retain the photoenzymatic activities of thylakoids and demonstrate the ability to drive an ATP-dependent enzymatic reaction both within and outside the modules under light irradiation. Specifically, we further elucidate the mechanisms underlying the prolonged activity conferred by microgel encapsulation, focusing on oxidation, protein and pigment degradation and structural disintegration of thylakoids. Our findings suggest the microgel-stabilized plant-derived energy module could work as a compartment for artificial cell design and photo-catalyzed ATP or protein synthesis.

## Materials and methods

### Materials

Sorbitol, 4-(2-hydroxyethyl)-1-piperazineethanesulfonic acid (HEPES), sodium L-ascorbate, 2,6-dichlorophenolindophenol (DPIP), K_2_HPO_4_, adenosine diphosphate (ADP), nicotinamide adenine dinucleotide phosphate (NADP), catalase, 3-morpholinopropanesulfonic acid (MOPS), sodium alginate, fluorescein 5-isothiocyanate (FITC), 3-(3,4-dichlorophenyl)-1,1-dimethylurea (DCMU) and luciferin were purchased from Aladdin. MgCl_2_ and sucrose were purchased from Tianjin Guangfu. Percoll was purchased from Yeasen. KOH and CaCl_2_ were purchased from Heowins. Ethylenediaminetetraacetic acid (EDTA) was purchased from Biofroxx. Dimethyl sulfoxide (DMSO) and acetone were purchased from Bohua. Bovine superoxide dismutase from bovine erythrocytes was purchased from Solarbio. Luciferase was purchased from Yuanye. Gelatin was purchased from Sigma.

### Isolation of thylakoids

Thylakoids were extracted from spinach by a modified method [21, 32]. Fresh spinach was purchased from local market and stored at 4°C in the dark overnight to consume the starch granules. Before use, the spinach leaves were washed three times by clean water and the main stems were removed by scissors. Then the leaves (50 g) and buffer 1 (50 mL; 330 mM sorbitol, 50 mM HEPES-KOH pH 7.6, 5 mM MgCl_2_, 0.1% (w/v) bovine serum albumin) were homogenized in a small blender. The homogenate was rapidly filtered through a 4-layer gauze and the filtrate was centrifuged at 4°C and 3000 × g for 10 min in a desk centrifuge (LX-155T500R, Haier). The pellet was resuspended by 25 mL buffer 2 (300 mM sorbitol, 50 mM HEPES-KOH pH 7.6, 5 mM MgCl_2_, 2 mM EDTA and 10 mM sodium L-ascorbate) and overlaid on a discontinuous Percoll gradients consisting of 7 mL 40% (v/v) percoll and 18 mL 80% (v/v) percoll solutions (80% (v/v) percoll, 10 mM sodium L-ascorbate, 300 mM sucrose, 66 mM MOPS-KOH pH 7.6; 40% (v/v) percoll, 10 mM sodium L-ascorbate, 300 mM sucrose, 25 mM MOPS-KOH pH 7.6). The gradients were centrifuged at 4°C and 3000 × g for 20 min. The factions containing intact chloroplasts and broken chloroplasts were pooled and resuspended in osmotic shock buffer (10 mM HEPES-KOH, 10 mM MgCl_2_, 10 mM sodium L-ascorbate). For long-term storage, 10% (v/v) DMSO was added to the thylakoid suspension, which was then frozen at −80°C.

### Quantification of chlorophyll

For naked thylakoid (NT), the suspension was subjected to a 200-fold dilution with 80% (v/v) acetone and then centrifuged at 10 000 × g for 10 min. For TM, the microgels were fully homogenized by a glass homogenizer. The homogenate was resuspended by 80% (v/v) acetone and then centrifuged at 10 000 × g for 10 min. The spectra of the supernatant were recorded between 600 and 700 nm in a closed cuvette by a spectrophotometer (UH5700, Hitachi). The absorbance was used to quantify the Chl (chlorophyll) according to the following formula:


$$\left[\mathrm{Chl}]=200\times (17.76\;A_{646.6}+7.34\;A_{663.6}\right)$$

where *[Chl]* (µg mL^−1^) is the concentration of Chl in the suspension, *A*_646.6_ is the absorbance at 646.6 nm and *A*_663.6_ is the absorbance at 663.6 nm [33].

### Optical characterization

The optical properties of samples were characterized by the spectrophotometer and a spectrofluorometer (Fluoromax-4, Horbia). The UV-Vis absorbance spectra scanning was set between 400 and 700 nm. For the fluorescence spectrophotometer, the excitation light was set at 436 nm and the emission spectra were detected between 600 and 800 nm.

### Evaluation of PSII activity

The PSII activity was assayed by the reduction of DPIP under light. The thylakoid suspension was centrifuged at 4°C and 3000 × g for 10 min to wash out sodium L-ascorbate and resuspended in reaction buffer solution (10 mM HEPES-KOH, 10 mM MgCl_2_, 100 μM DPIP). For microgels, DPIP was added to the stock solution to generate microgels containing 200 μM DPIP. The microgels were diluted 1:1 in osmotic shock buffer without sodium L-ascorbate. The reaction buffer solution was added to a 96-well plate with 200 μL each well and the absorbance at 600 nm was recorded by a microplate reader (Varioskan Flash, Thermo Fisher) every 10 s.

### Evaluation of ATP production under light

The ATP production was assayed in a reaction buffer solution (50 mM HEPES-KOH pH 7.6, 10 mM sodium L-ascorbate, 5 mM MgCl_2_, 5 mM K_2_HPO_4_, 10 mM KCl, 2 mM ADP, 3 mM NADP, 1.5 μM catalase and 52 U mL^−1^ of bovine superoxide dismutase). The reaction buffer solution was added to a 96-well plate with 200 μL each well to receive illumination evenly. A xenon lamp (Wence, WC-150) with adjustable power was used as the light source. After illumination, the reaction buffer solution was centrifuged at 4°C and 12 000 × g for 5 min and the concentration of ATP in the supernatant was measured by an assay kit (Beyotime, S0026).

### Design and fabrication of the micromold

A stainless steel array chip (75 mm × 25 mm × 200 μm) was used as a micromold. The array chip was designed by Solidworks 2019 software and fabricated by a laser cutting machine. The array contained 150 × 50 circular holes with an identical radius of 200 μm. To enhance the adhesion between the chip surface and the precursor solution, the chips were treated with treated with Plasma Cleaner (Diener, Atto-BR-200-PCCE) for 5 min.

### Fabrication of microgels by the micromold

Different amounts of equal sodium alginate and gelatin were fully dissolved in osmotic shock solution to gain precursor solution with various mass concentrations (1 wt%, 2 wt%, 4 wt%). About 100-μL precursor solution was pipetted to the surface of the micromold and filled every circular hole with the help of a cover slide. The cover slide scraped the precursor solution forth and back for several times to evenly distribute the solution and remove excessive solution in case of the formation of irregular microgels. Next, the chip was cooled down at 4°C at a refrigerator for 1 min to preliminarily fix the shape of microgels. After that, the chip was immersed into pre-cooled 25 mM CaCl_2_ solution for 30 s to form strong crosslinks and immediately removed off. The chip was subsequently treated with an ultrasonic cleaner for 4 min. Eventually, the microgels were collected by centrifuging and filtering through a 70 μm cell strainer.

### Rheological test

The rheological property of microgels were assayed by an advanced rotary rheometer (MCR302, Anton Paar) was applied to characterize the rheology of the gels. The angular frequency sweep was investigated with the frequency ranging from 0.1 to 100 rad s^−1^ at a constant strain of 1%. The strain sweep was carried out by changing strain value from 0.1% to 100% at a fixed angular frequency of 1 rad s^−1^.

### Visualization of microgels

In order to visualize the microgels, gelatin was engrafted with FITC by adding 400 μg FITC into 10 mL precursor solution and then stirring for 4 h. The microgels were observed by an inverted fluorescence microscope (DMi8, Leica) in bright field, red channel and green channel. The sizes of microgels were measured by ImageJ software.

### Evaluation of chlorophyll degradation

At the first day, NT and TM containing the same content of Chl were produced by the quantitative method mentioned above. Then the NT and TM were stored together on ice in the dark. At every time point (0, 3, 7 and 21 days), the Chl contents were quantified. For visualization of Chl concentration, the acetone extraction solutions were centrifuged at 10 000 × g for 10 min and the shades of supernatant were captured in pictures by a camera.

### Western blotting

For NT suspension, RIPA buffer (R0020, Solarbio) was added into the suspension and incubated on ice for 30 min to release proteins from the thylakoids. For TM, the microgels were fully homogenized by the glass homogenizer before addition of RIPA buffer and incubation. After incubation, the samples were centrifuged at 4°C and 12 000 rpm for 15 min to gain the supernatant containing released proteins. These proteins were separated by SDS–PAGE and then transferred to PVDF membrane. Anti-PsbA/D1 rabbit antibody (PAB02001, Orizymes; 1:3000) and Anti-PsbD/D2 rabbit antibody (PAB02002, Orizymes; 1:3000) were chosen as the primary antibodies, and horseradish peroxidase-linked anti-rabbit IgG antibody (7074P2, Cell Signaling; 1:2000) was chose as the secondary antibody. The PVDF membrane was visualized by a chemiluminescence machine (Tanon, 5200) after incubated with enhanced chemiluminescence western blotting substrate (Solarbio, PE0010). These steps were repeated at every preservation time point. The quantitative analysis was accomplished by ImageJ software.

### Evaluation of thylakoid membrane disintegration

Glutaraldehyde fixed solution was added into the sample (NT and TM stored for different time) to preserve the structure of thylakoid membrane. The samples were cut into thin sections after a series of processes and pictures were captured by a transmission electron microscope (TEM). The quantitative analysis was accomplished by ImageJ software.

### Evaluation of TM to drive an ATP-dependent enzymatic reaction

In order to construct an energy supply subunit based on TM and test its ability of driving the luciferin/luciferase reaction, ADP, luciferin and luciferase were added into the precursor solution. The TM was diluted by osmotic shock solution to create a reaction system finally containing 2 mM ADP, 1.4 mg ml^−1^ luciferin, 0.2 mg mL^−1^ luciferase and 20 µg mL^−1^ Chl. The reaction system was illuminated by a beam of white light at an intensity of 25 W m^−2^ and the luminescence intensity was measured by the microplate reader every 4 min.

In order to visualize the process of the luciferin/luciferase reaction, 10 µL of reaction system liquid was pipetted onto a glass slide and observed by the inverted fluorescence microscope in a dark room. The glass slide was illuminated under a beam of white light at an intensity of 12.5 W m^−2^ and the photos in green and red channels were captured every 10 min with the same exposure parameters. The colocalization analysis between red signals and green signals was accomplished by ImageJ software.

### CCK-8

L929 cells were bought from iCell. The cells were co-cultured with RPMI 1640 medium (Gibco, 11875093) containing various volume fractions of TM (3‰, 6‰, 12‰, 25‰, 50‰, 100‰ and 200‰) in 96-well plates for 24 and 48 h. The cells were washed with PBS and subsequently co-cultured with CCK-8 solution (C0038, Beyotime) at 37°C in a CO_2_ incubator (HF240, Heal Force) for 2 h. The absorbance at 450 nm was measured by the plate reader. The cell viability was calculated by the following formula:


$$\mathrm{Cell}\;\mathrm{viability}\;(\%)=\frac{{\mathrm{Abs}}_{E}-{\mathrm{Abs}}_{\mathrm{CK}}}{{\mathrm{Abs}}_{C}-{\mathrm{Abs}}_{\mathrm{CK}}}\times 100\%$$

where Abs_E_ is the absorbance of the experiment groups (co-cultured with RPMI 1640 containing TM) at 450 nm, Abs_CK_ is the absorbance of the CCK-8 solution at 450 nm and Abs_C_ is the absorbance of the control group (co-cultured with RPMI 1640 only) solution at 450 nm.

### Live/dead staining

L929 cells were co-cultured with RPMI 1640 medium containing various volume fractions of TM (3‰, 6‰, 12‰, 25‰ and 50‰) in 24-well plates for 24 h. The cells were washed with PBS and subsequently co-cultured with dead/live staining solution (C2015M, Beyotime) in the dark at 37°C in a constant temperature incubator (QE-2, Honour) for 30 min. The inverted fluorescence microscope was used to capture photos in red and green channels. The photos were then utilized to count the numbers of dead cells and live cells by ImageJ software. The live cell ratio was calculated by the following formula:


$$\mathrm{Live}\;\mathrm{cell}\;\mathrm{ratio}\;(\%)=\frac{N_{L}}{N_{L}+N_{D}}\times 100\%$$

where *N*_L_ is the number of live cells in a photo, and *N*_D_ is the number of dead cells in the same photo.

## Results

### Preparation and characterization of microgel-encapsulated thylakoid energy modules

**Figure 2:**
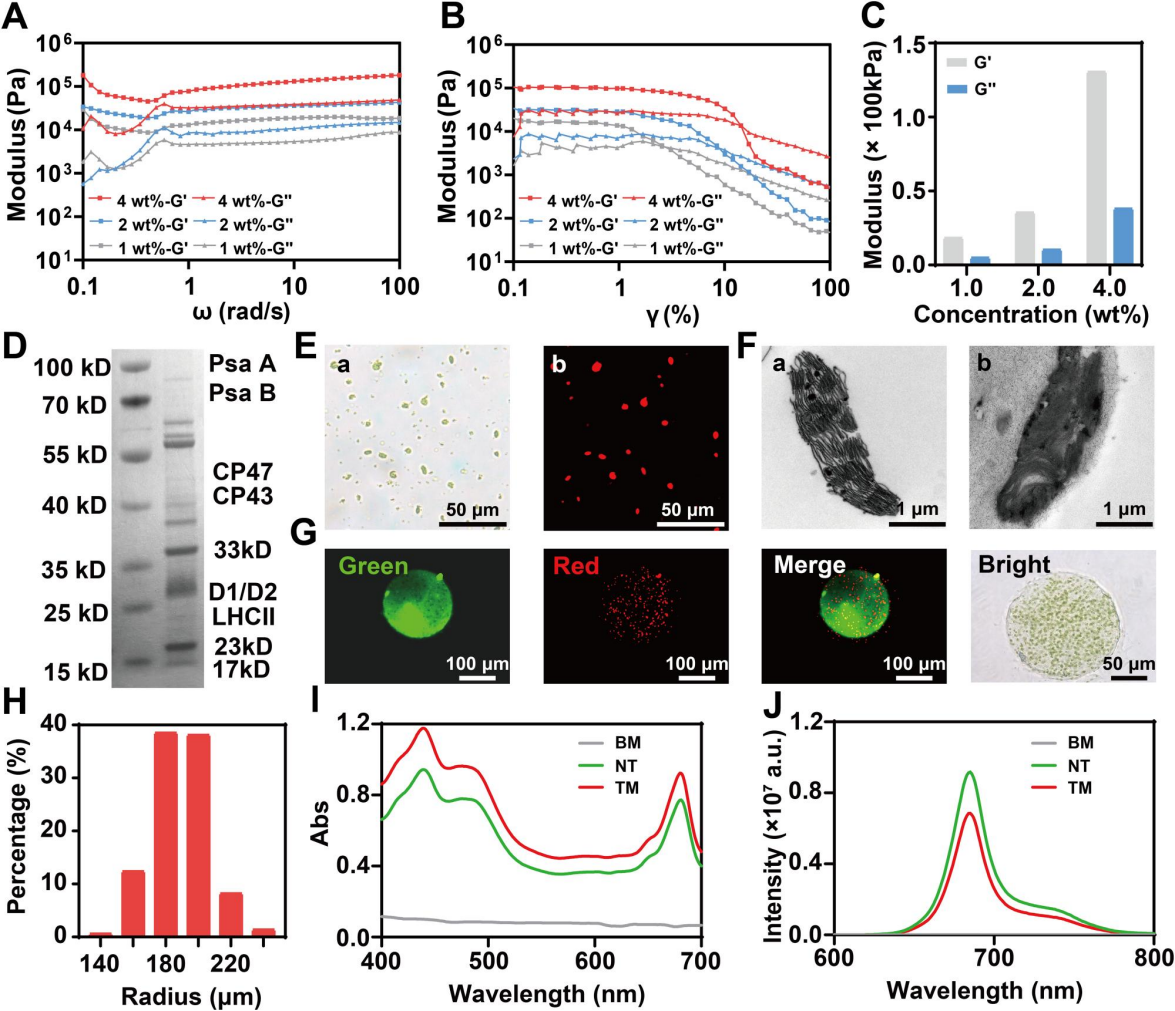
Preparation and characterization of TM. (**A**) Rheological analysis of TM (angular frequency sweep scanning). (**B**) Rheological analysis of TM (strain sweep scanning). (**C**) Modulus of TM at various concentrations measured under an angular frequency of 10 rad s^−1^ and a strain of 1%. (**D**) SDS–PAGE results: the left lane shows the marker, and the right lane shows the sample protein bands. (**E**) Morphology of NT observed under fluorescence microscopy: (a) bright field and (b) red channel. (**F**) Morphology of NT observed under TEM: (a) NT and (b) TM. (**G**) Morphology of TM observed under fluorescence microscopy: green channel, red channel, merge, and bright field. (**H**) Radius distribution of TM. (**I**) UV-Vis absorption spectra of TM, NT and blank microgel (BM). (**J**) Fluorescence spectra of TM, NT and BM under 436 nm light excitation.

We mixed sodium alginate and gelatin in equal mass fractions to generate Alg/Gel microgels as the delivery capsule for our energy supply modules. The microgels were fabricated through a previously reported micromold method with some modifications [34]. The micromold was designed as a stainless steel chip with an array consisting of thousands of circular holes. Each hole had a diameter of 200 μm and a depth of 200 μm to precisely determine the size of microgels (Supplementary Figure S1). In the initial step, the chip was evenly scraped with an Alg/Gel precursor solution to ensure every hole was fully filled. Then the chip was cooled at 4°C and subsequently immersed into CaCl_2_ solution (25 mM) for gelation (Supplementary Figure S2). The addition of gelatin could increase the viscosity of the precursor solution, thus preventing it from flowing off the holes. Furthermore, the gelatin could form a weak hydrogel upon cooling (Supplementary Figure S3) to preliminarily fix the shape of microgels before a strong crosslink formed between alginate and calcium ion. Even though previous research had demonstrated that low concentrations of calcium ions have little impact on PSII’s activity [35], we still minimized the contacting time of CaCl_2_ solution to avoid possible damage. By using this method, we produced a large quantity of microgels rapidly (∼50 μL microgels per chip per production cycle, 5 min per production cycle). We prepared three different precursor solutions with gradient mass concentrations of 1 wt%, 2 wt% and 4 wt%, and tested their rheological properties via angular frequency and strain sweep scanning (Figure 2A and B). As the precursor concentration increased, the modulus of the microgels was increased (Figure 2C), yet they exhibited similar mechanical behaviors, such as shear-thinning.

Thylakoids were successfully extracted from fresh spinach leaves by a modified method (Supplementary Figure S4) involving homogenization, filtration, centrifugation and osmotic shock [21, 32]. After isolation, a series of characterizations were carried out to verify the extract was thylakoid. Proteins in the extract were analyzed through SDS–PAGE and coomassie blue staining (Figure 2D). The bands of typical proteins associated with photosynthesis demonstrated the presence of PSII [36]. Under an optical microscope, numerous green short rod-like structures were observed in the bright field, which emitted red fluorescence under excitation by blue light, due to the fluorescence properties of Chl (Figure 2E). TEM image revealed the typical thylakoid membrane structure and showed that the outer and inner membranes of chloroplasts were disrupted in the process of osmotic shock (Figure 2F). Additionally, the differences between grana thylakoids (stacked membranes) and stroma thylakoids (membranes connecting between grana) could be clearly distinguished. These characterization results indicated the successful isolation of thylakoids from spinach.

We subsequently encapsulated thylakoids within Alg/Gel microgels to prepare TM, and investigated whether the operation of encapsulation would affect the physicochemical properties of thylakoids. The top morphology of the microgel exhibited a circular shape with a radius around 200 μm (Figure 2G), which was consistent with the dimensions of the circular holes in the micromold. Thylakoids were uniformly distributed throughout the microgel. To further evaluate the morphology of the microgels, gelatin was grafted with a green fluorescence dye, FITC, and was observed under multiple channels. The statistic result of radius measurement indicated that micromold ensured the radius of TM was concentrated within a narrow range (Figure 2H). To quantify the thylakoids, we measured the absorbance of the extract and employed a revised version of Arnon’s equation to calculate the concentration of Chl [33]. The UV-Vis absorption spectrum exhibited three characteristic peaks around 430, 470, and 660 nm (Figure 2I), indicating that the extract was rich in two essential chloroplast pigments, Chl a and Chl b. The peak at 670 nm in the fluorescence spectrum under 436 nm excitation (Figure 2J) further verified the presence of chloroplast pigments [37]. Compared with NT, TM containing the same amount of Chl presented a similar morphology but a higher baseline in UV-Vis absorption spectrum due to the absorption of Alg/Gel microgels. Nevertheless, the fluorescence intensity underwent a significant reduction after encapsulation, because microgels might shelter the fluorescence. Furthermore, the process of encapsulation showed minimal influence on the structure of thylakoids as the encapsulated thylakoid membranes remained basically intact (Figure 2F). We also investigated the stability of TM by quantifying thylakoid release (Supplementary Figure S5). After storage at room temperature for 7 days, only ∼3% of encapsulated thylakoids was lost, indicating that microgels could serve as compartments to spatially confine these membrane structures.

**Figure 3:**
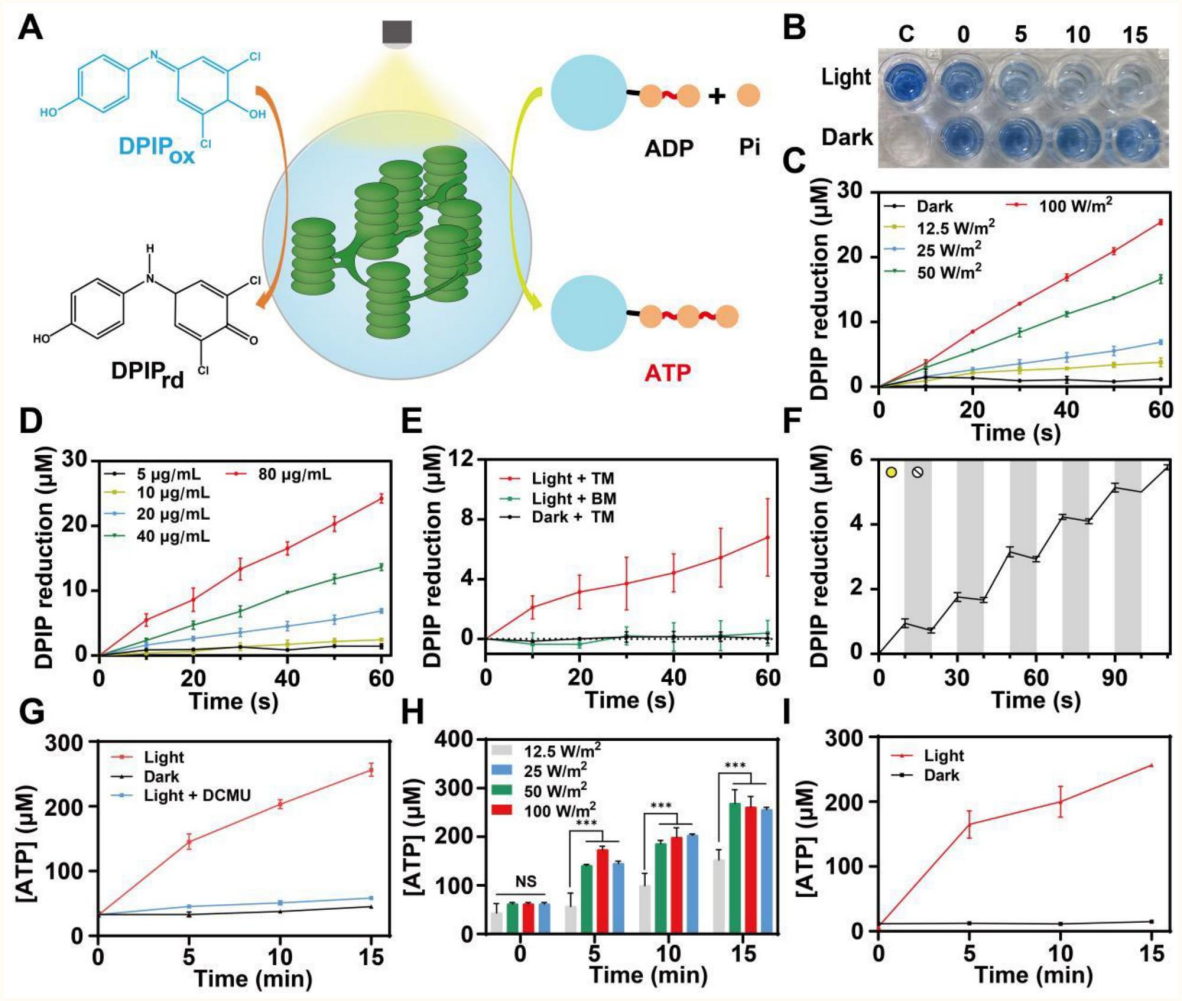
Photoenzymatic process assay of NT and TM. (**A**) Scheme illustration of assaying TM’s photoenzymatic process. (**B**) Morphology of DPIP solution mixed with NT under light at different time points. (**C**) DPIP reduction by NT under light with increasing intensity (ΔAbs was defined as the difference between the initial absorbance value and the absorbance values at subsequent time points; *n* = 3, mean±SD). (**D**) DPIP reduction by various concentrations of NT under white light (the light intensity was 25 W m^−2^; *n* = 3, mean±SD). (**E**) DPIP reduction by TM (20 μg ml^−1^ Chl equivalent) under white light (the light intensity was 25 W m^−2^; *n* = 3, mean±SD). (**F**) DPIP reduction by TM (20 μg ml^−1^ Chl equivalent) under pulsed white light (the light intensity was 25 W m^−2^; *n* = 3, mean±SD). (**G**) ATP production by NT (15 μg ml^−1^ Chl equivalent) under white light (the light intensity was 25 W m^−2^; *n* = 3, mean±SD). (**H**) ATP production by NT (15 μg ml^−1^ Chl equivalent) under white light with increasing intensity (*n* = 3, mean±SD). (**I**) ATP production by TM measured via quantifying ATP concentrations in the reaction buffers (15 μg ml^−1^ Chl equivalent) under white light (the light intensity was 25 W m^−2^, *n* = 5, mean±SD). ***P < 0.001, NS (no significance): *P* > 0.005. *P*-values were calculated by one-way analysis of variance (ANOVA).

The thylakoid contains the proteins, pigments and co-factors involved in light reactions. Briefly, PSII captures light energy and utilizes it to extract electrons from water, generating oxygen as a byproduct. The electrons are subsequently transferred to photosystem I (PSI) through plastoquinone and cytochrome b6f and eventually donated to the oxidized form of NADP, reducing it to the reduced form of nicotinamide adenine dinucleotide phosphate (NADPH). During the electron transfer, a proton concentration gradient, which drives the production of ATP via ATP synthases, is established across the thylakoid membrane [38]. Therefore, we selected the activity of PSII and ATP production capacity, which represent the initiation and termination of photosynthetic light reactions occurring on the thylakoid membrane, respectively, to assess and compare the activities of NT and TM (Figure 3A).

An artificial electron receptor, DPIP, was utilized as an indicator for the electron transfer capacity of PSII. After receiving electrons from PSII, DPIP can be reduced, resulting in a color change from blue to colorless. We mixed DPIP and NT suspension to create a reaction system containing 40 μg mL^−1^ Chl and 60 μg mL^−1^ DPIP. Under illumination at a light intensity of 100 W m^−2^, the blue color was observed visibly lighter, suggesting the reduction of DPIP. By contrast, the color remained essentially unchanged in the dark (Figure 3B). Compared to the control group without NT, the color of both the light and dark groups at 0 min was slightly lighter. This phenomenon might be attributed to the existence of intrinsic reducing substances within NT, such as the terminal iron-sulfur cluster of PSI [39]. To quantify the kinetics of DPIP reduction by PSII, the absorbance changes at 600 nm were recorded and converted into the concentration of reduced DPIP. Maintaining the contents of Chl and DPIP at 20 and 30 μg ml^−1^, respectively, we evaluated the DPIP reduction kinetics under incremental light intensities ranging from 0 W m^−2^ (dark) to 100 W m^−2^. The amount of reduced DPIP increased linearly with time, and the reduction rate presented a positive correlation with light intensity (Figure 3C). Similarly, when the light intensity was fixed at 25 W m^−2^ and the Chl content was increased from 5 to 80 μg mL^−1^, we observed a comparable trend (Figure 3D).

Next, we tested the capacity of PSII in TM to transfer electrons under illumination. The isolated thylakoids and DPIP were co-encapsulated in TM and suspended in a buffer solution to establish a reaction system containing 20 μg mL^−1^ Chl and 30 μg ml^−1^ DPIP. When exposed to a beam of white light at a constant intensity of 25 W m^−2^, the TM system exhibited DPIP reduction kinetics similar to those of NT (Figure 3E). The DPIP reduction rate of TM (6.76 ± 2.02 μM min^−1^) was comparable to that of NT (6.89 ± 0.27 μM min^−1^) when they contained the same Chl content, indicating that the activity of PSII was not affected after encapsulation. Moreover, under pulsed light at 10-s intervals, the DPIP reduction in the TM system showed a stepwise increase (Figure 3F). The reduction of DPIP only occupied during the illumination interval and eased immediately once the light was turned off. These results suggested that the overall activity of TM could be precisely activated or deactivated by switching between light and dark, as the photosynthesis was initiated by PSII.

Following the preliminary confirmation of the upstream photosynthetic activity of NT and TM, their ATP-producing capacity was assessed. Under white light at an intensity of 25 W m^−2^, the ATP concentration in the NT system increased from 31.8 ± 0.7 to 256.2 ± 3.4 μM within 15 min (Figure 3G). In the absence of illumination, only moderate ATP production of NT was observed, averaging 12.3 μM. The addition of a herbicide, DCMU, which specifically inhibits PSII and disrupts the formation of the proton concentration gradient [40], significantly reduced ATP production of NT under illumination (25.6 μM vs. 224.4 μM). These findings collectively supported that the ATP production was mainly from the photosynthetic processes initiated by PSII. The moderate ATP production in the dark may be attributed to the membrane-bound adenylate kinases [41]. By subtracting the contribution of adenylate kinases, we normalized the ATP production rate to 4.7± 0.8 μM min^−1 ^μg^−1^ Chl. Additionally, the ATP production within 15 min at various light intensity (12.5, 25, 50 and 100 W m^−2^) was measured (Figure 3H). When the light intensity exceeded 25 W m^−2^, the ATP production plateaued, with no significant difference in total ATP yield at 25, 50 and 100 W m^−2^. This consistency, which could be explained by the light saturation limiting the regeneration of cofactors, aligns with previous research findings [21]. To balance photosynthetic efficiency and photooxidation, we chose 25 W m^−2^ as the light intensity for the following experiments. Subsequently, we measured the ATP-producing capacity of TM, which was comparable to that of NT (Figure 3I). In addition, this consistency was further validated in TMs with various precursor concentrations (Supplementary Figure S6), indicating that the microgels not only had little negative effect on thylakoids’ activity but also fully outputted the produced ATP to external environment. Additionally, the production of another product during photoenzymatic processes, NADPH, was assayed (Supplementary Figure S7). While NADPH production of TM was higher than that of NT, perhaps due to the molecular crowding effect, the NADPH level in both groups was relatively low compared with ATP production. Given that the initiation and termination of photosynthetic light reactions were both unaffected, it can be concluded that TM effectively retains the light reaction components and inherits the photosynthetic activity from thylakoids.

### Microgel encapsulation provides thylakoids with prolonged activity

The preservation of activity plays a paramount role in the development of artificial cells and artificial organelles. It is essential to extend the effective working period or to mimic the lifetime of natural cells. Therefore, we investigated the activity duration of TM by measuring PSII activity and ATP production and attempted to elucidate the mechanisms underlying the protective efficacy of microgel encapsulation.

**Figure 4:**
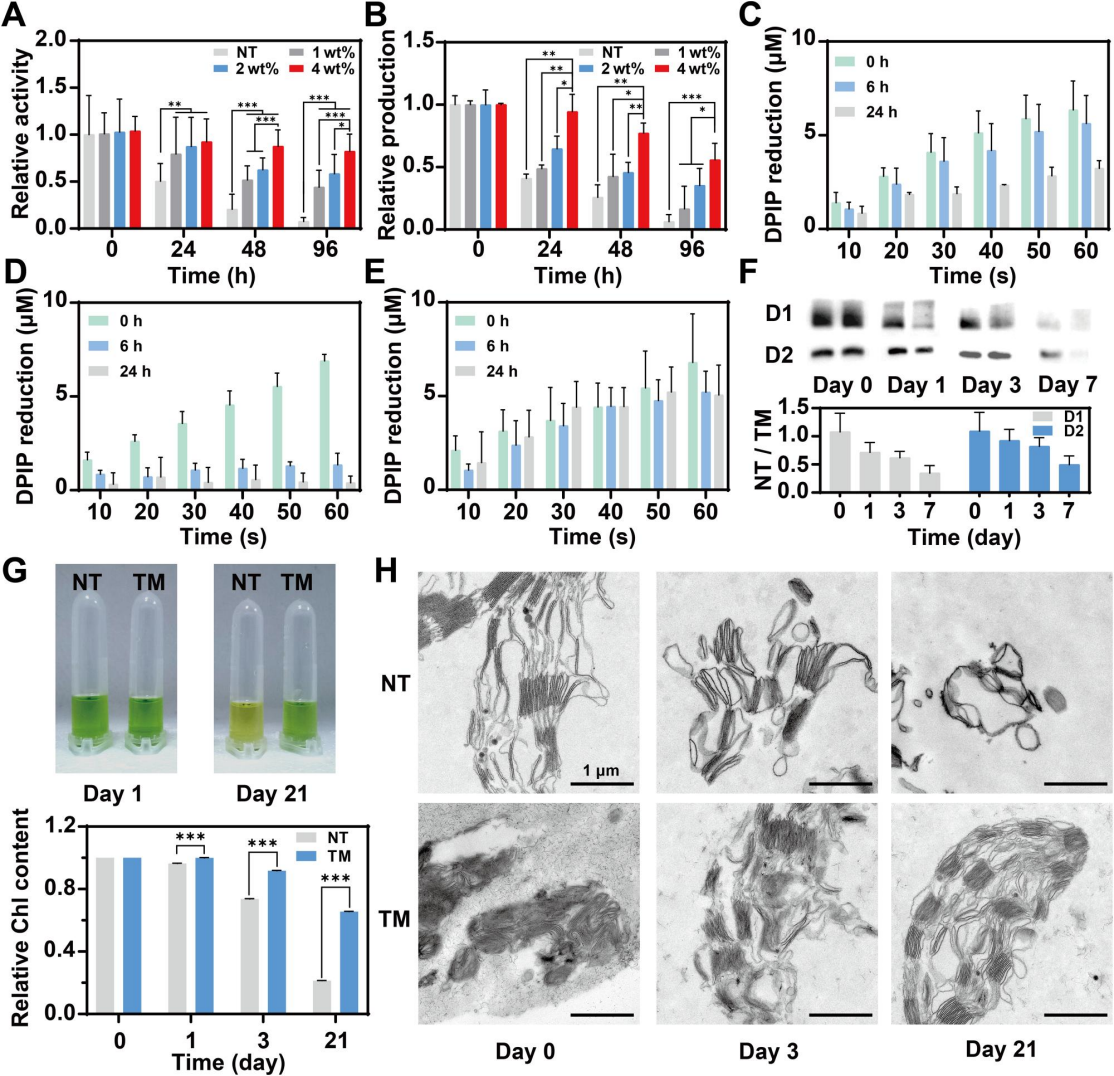
Prolonged activity of TM and the underlying mechanisms. (**A**) Changes of PSII’s relative activity over time in NT and TM with precursor concentrations of 1 wt%, 2 wt% and 4 wt% (relative activity was defined as the ratio between the DPIP reducing capacity of the samples at each time point and that of freshly isolated NT after isolation; the light intensity was 25 W m^−2^; *n* = 12, mean±SD). (**B**) Changes of ATP relative production over time in NT and TM (the ATP production of TM was measured via quantifying ATP concentrations in the reaction buffers; the light intensity was 25 W m^−2^; *n* = 3, mean±SD). (**C**) Changes of DPIP reduction kinetics over 24 h in NT stored with antioxidants (the light intensity was 25 W m^−2^; *n* = 3, mean±SD). (**D**) Changes of DPIP reduction kinetics over 24 h in NT stored without antioxidants (the light intensity was 25 W m^−2^; *n* = 3, mean±SD). (**E**) Changes of DPIP reduction kinetics over 24 h in TM stored without antioxidants (the light intensity was 25 W m^−2^; *n* = 3, mean±SD). (**F**) Representative pictures of western-blotting results for D1 and D2 subunits and their content ratio between NT and TM via semi-quantitative analysis (for each time point, the right lane was TM and the left lane was NT; NT/TM was defined as the ratio of NT’s and TM’s gray value from the same PVDF membrane; *n* = 6, mean±SD). (**G**) Chl degradation of NT and TM over time (*n* = 3, mean±SD). (**H**) Morphology of thylakoid membranes in NT and TM observed by TEM. *P < 0.05, **P < 0.01, ***P < 0.001, NS (no significance): *P* > 0.005. *P*-values were calculated by Student’s *t*-test or ANOVA.

We constructed three types of TMs with various concentrations of precursor (1 wt%, 2 wt% and 4 wt%) mentioned above and compared their abilities to reduce DPIP under illumination with that of NT. Between the measuring time points, both NT and TM were stored at 4°C in the dark to avoid protein damage caused by photooxidation and high temperature [42, 43]. After immediate encapsulation, the activities of the three TMs were comparable to that of NT (Figure 4A). At 24 h after encapsulation, the activity of NT decreased to half of its original level, while all TMs retained at least 79.4% of PSII’s function. Furthermore, this preservative efficacy was positively correlated to the precursor concentration of TM. By 96 h after encapsulation, the TM made by 4 wt% Alg/Gel preserved 82.2% activity while that for TM made by 1 wt% Alg/Gel was 44.1%. Thus, we selected the TM made by 4 wt% Alg/Gel in the following experiments. As shown in Figure 4B, after being stored for 96 h, TM exhibited a notably higher ATP production level and the relative ATP production could retain up to 55.8% of its initial level, while NT lost 94.7%. Moreover, the retention of ATP production was correlated with the concentration of microgel precursors, which was similar to that of PSII activity. Such a phenomenon might be explained by the functional mechanisms of ATP synthase, as it is driven by a proton concentration gradient. The formation of the gradient was initiated by the photolysis of water in PSII. Therefore, it is reasonable to conclude that ATP production capacity and PSII activity exhibited a certain degree of consistency in their retention. Furthermore, the ATP production also involved other protein complexes, co-factors and enzymes located across the thylakoid membrane. Given the collective decline in activity of all these components, the reduction in ATP production capacity was significantly more pronounced than that in PSII activity.

Then, we explored the mechanisms underlying the preservative efficacy of microgel encapsulation from the perspectives of shielding oxidation, inhibiting protein and Chl degradation, and maintaining structural integrity. During the isolation processes of organelles, such as mitochondria and chloroplasts, antioxidants were typically added into the isolation buffer to prevent oxidative damage caused by oxygen or other reactive species in extracellular environments [23, 44]. Here, we added sodium L-ascorbate (also known as vitamin C) into the isolation buffer and storage buffer. To evaluate the role of the microgel encapsulation in anti-oxidation, we removed sodium L-ascorbate from the buffers and measured the PSII activities of TM and NT within 24 h. The removal of the antioxidant caused a more rapid decline of PSII activity in the NT group than that in the TM group (Figure 4C and D). At 6 h after isolation, the NT under the protection of sodium L-ascorbate maintained 87.23% activity, while the activity proportion of the NT without antioxidant fell to 20%. By 24 h, this gap continued to widen. However, the removal of antioxidant showed smaller impact on TM’s PSII activity (Figure 4E). The preserved activity of TM with or without antioxidant at 24 h were 92.5% and 74.8%, respectively, suggesting that microgels could shield oxidation.

We subsequently evaluated the protein degradation using a semi-quantitative method based on western blotting. Two key subunits of PSII, D1 and D2, were selected as biomarkers to represent the total protein degradation dynamics. To ensure the comparability, NT and TM samples were analyzed on the same western-blotting membrane (Figure 4F). The ratio of the gray values of NT to TM was calculated to compare their non-degraded protein levels. During storage, the disparity in protein content between NT and TM progressively increased. By Day 7, the NT/TM ratio for D1 and D2 decreased to 0.34 and 0.49, respectively, demonstrating TM’s ability to mitigate protein degradation. Parallel to protein degradation analysis, we also assessed the degradation of Chl. The results demonstrated that TM’s superiority in preserving Chl became significant at Day 1 post-encapsulation (Figure 4G). At Day 3, Chl concentration in NT group was 73.6% of the initial one, whereas that for TM was 91.7%. By Day 21, visual distinction emerged between the two groups (21.4% vs. 65.5%). These findings conclusively demonstrate that TM significantly delays both the protein and Chl degradation.

Finally, to investigate structural preservation, we immobilized NT and TM membrane structures at designated time points and observed their structural integrity through TEM. Fresh isolated spinach thylakoid membranes exhibited native architecture, characterized by well-organized stroma and grana thylakoids with intact lateral connections (Figure 4H). In NT samples, however, the normal configuration of thylakoid membrane progressively transformed into a disordered state, where the quantity of broken thylakoid pieces increased significantly and the lumen constantly swelled (Supplementary Figure S8). This degenerative process culminated in severe membrane disintegration by Day 3 with 7.4 ± 3.2 broken thylakoids per view and an average lumen area of 0.011 μm^2^ compared to 0.005 ± 0.002 μm^2^ for fresh thylakoid lumen. In stark contrast, the grana thylakoids in TM remained basically intact with 1.0 ± 0.6 broken thylakoids per view and only exhibited a slight deformation with an average lumen area of 0.007 μm^2^ by Day 3, which might be caused by swelling [45]. By Day 21, the gaps between NT and TM were more significantly pronounced in the number of broken thylakoids per view (8.8 ± 2.5 in NT vs. 2.0 ± 1.2 in TM) and the average lumen area (0.069 μm^2^ in NT vs. 0.007 μm^2^ in TM). Based on these observations, TM was capable of preventing the disintegration and deformation of thylakoid membrane during storage.

In summary, the prolonged functional retention of TM stems from the protection mechanisms in three aspects: (i) oxidation shielding effect, (ii) inhibition of protein and Chl degradation and (iii) stabilization of membrane structure.

Our mechanistic analysis reveals that enhanced preservative efficacy in TM with higher precursor concentrations is directly linked to microgel network porosity. Typically, elevated precursor concentrations yield denser polymer networks, resulting in the restriction of molecular exchange across the gel–environment interface [46]. This view aligned with our scanning electron microscope observations. After freeze drying, the TM made of 1 wt% and 2 wt% Alg/Gel showed relatively higher porosity while few visual pores and significantly lower porosity were observed on the surface of TM made of 4 wt% Alg/Gel (Supplementary Figure S9). We proposed that the microgel impeded the diffusion of hazardous oxidative species, such as external oxygen, residual ROS and phenolic compounds, thereby shielding bioactive components from damage. Similar oxidation shielding effect was also previously reported by Picone *et al.*, who developed a degalactosylated xyloglucan hydrogel that could protect the loaded mitochondria from H_2_O_2_ [31]. The reduced oxidative stress might contribute to a lower degradation speed of proteins and Chl [46, 47], thus prolonging the lifetime of TM’s activity. Oxidative agents could scissor peptide bonds and cleave the hydrophilic portion of the NH_(2)_ region, thereby leading to fragmentation of photosynthesis-related proteins (e.g. D1 and D2) [48, 49]. Although this damage naturally occurs during photooxidation under strong illumination, it also happens in darkness when PSII is treated with ROS [50]. The oxidative agents also trigger Chl degradation by oxidizing Chl to colorless low molecular weight compounds [51]. Therefore, microgel network might impede protein and Chl degradation by shielding external oxidative agents.

To elucidate the shielding effect caused by the restricted diffusion within the microgel networks, we investigated the activity changes of various TMs in a reaction system containing 100 μM DCMU (Supplementary Figure S10). Our results revealed a concentration-dependent enhancement of PSII activity with retention up to 56.6 ± 17.1%, while the naked NT without protection only retained 8.8 ± 5.4% of its activity. By contrast, after DCMU was encapsulated into the microgels, all of TMs showed a similar activity reduction, suggesting that the activity retention was attributed to the microgel networks, which effectively hindered the penetration of DCMU into the microgel matrix.

In addition to proteins and Chl, the restricted diffusion may also prevent rapid oxidization of the membrane lipids to mitigate subsequent fluidity reduction and structure disintegration, which are essential to photosynthesis, as membrane’s intact structure and normal fluidity ensure the stable interactions within protein complexes and the formation of the proton gradient [42]. Moreover, hydrogel encapsulation can also stabilize lipid membranes via generating a double-crosslinked framework based on weak physical interactions (e.g. hydrogen bond) between them [52].

### Validation of the artificial light-controlled energy supply module based on TM

**Figure 5:**
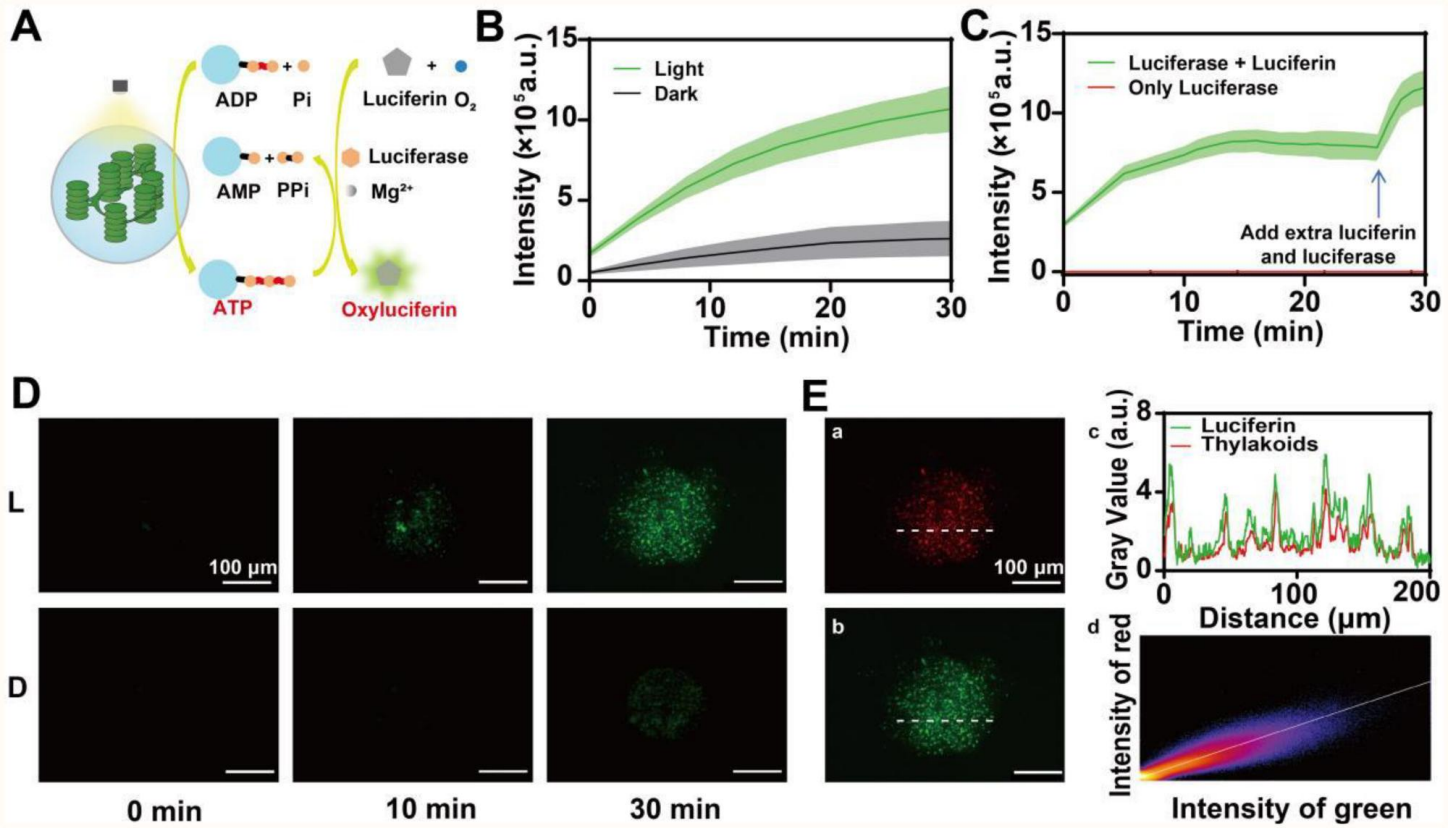
Validation and cytocompatibility of the artificial light-controlled energy supply modules. (**A**) Scheme illustration of energy supply modules to drive luciferin/luciferase enzymatic process under light. (**B**) Luminescence intensity changes of the energy supply modules under light and in the dark (the light intensity was 25 W m^−2^; *n* = 3, mean±SD). (**C**) Luminescence intensity changes of the energy supply modules with half of luciferin and luciferase added at 25 min or without luciferin (the light intensity was 25 W m^−2^; *n* = 3, mean±SD). (**D**) Morphological changes of a single energy supply module under light and in the dark (marked as ‘L’ and ‘D’, respectively). (**E**) Co-localization analysis of the emitting oxyluciferin (green) and thylakoids (red): (a) morphology observed in the red channel and (b) morphology observed in the green channel, (c) signal intensity line graph and (d) coincidence 2D graph of green and red signals. The data in the line graph was gained from the slices in sub-picture (a) and (b).

After confirming the long-term retention of TM activity, we validated our artificial light-controlled energy supply module by testing whether it could drive an ATP-dependent enzymatic process. Firefly luciferase, a natural enzyme discovered in fireflies, has been demonstrated to catalyze the oxidization of luciferin to emit yellow-green luminescence. This reaction is powered by ATP in the presence of O_2_ and Mg^2+^ [53]. Therefore, we chose the luciferin/lucierase enzymatic system to validate the energy-supplying capacity of TM (Figure 5A).

During the fabrication, we encapsulated luciferin and luciferase within the module by adding them into the precursor solution. Then we suspended TM in a reaction solution containing ADP, phosphoric acid (Pi) and Mg^2+^. The O_2_ was supplied by the dissolved oxygen in the solution. Under white light illumination, luminescence was detected and its intensity increased over time, while the luminescence in the dark was significantly weaker with only a moderate increase over time (Figure 5B). When the luciferin was removed from the module, no luminescence signal was measured throughout the illumination process (Figure 5C), suggesting the luminescence signal came from the successfully driven reaction by the ATP from TM rather than the excited luminescence from Chl. Intriguingly, when we halved the luciferin and luciferase content encapsulated in the TM, the luminescence intensity remained basically unchanged at 15 min, suggesting that the reaction reached a balanced state. After the other half of luciferin and luciferase was directly added into the solution, the reaction equilibrium shifted in the forward direction and the luminescence intensity rose to a level comparable to that in the previous reaction system with the original luciferin content. These data indicated that the excessively generated ATP diffused outside TM and successfully powered the other half reaction system as an energy output.

The luminescence could be observed visually under an inverted microscopy. We tracked the changes of a single module over time by capturing pictures with the same exposure parameter in the green channel. Under illumination, the green luminescence signal progressively increased in intensity over time and exhibited multiple short rod-like structures limited within a circular area (Figure 5D). For the modules in the dark, a similar phenomenon was observed but the luminescence intensity was significantly lower compared to that of the illuminated ones. Additionally, we calculated the Pearson’s correlation coefficient between the green and red signals within the same module, and discovered that these two signals were highly coincident (Pearson’s *R* value (no threshold) = 0.92) (Figure 5E). If we assumed that luciferin and luciferase were evenly distributed within the module, the intensity of green luminescence signal could be regarded as an indicator of ATP concentration, which was significantly higher around thylakoids. Our analysis further demonstrated that the ATP was synthesized by thylakoids and drove surrounding enzymatic processes by diffusion. Moreover, this reaction system could function persistently for 96 h, with the maximal luminescence intensity retaining half of the original level (Supplementary Figure S11). This was facilitated by the long-lived ATP production capacity of the energy supply modules, which provided a robust power source for the enzymatic reaction.

To assess the cytocompatibility of TM, L929 cells were co-cultured in the RPMI 1640 medium with increasing volume fractions of TM (from 3‰ to 20‰) for 24 and 48 h. When the TM volume fractions were below 50 ‰, the results of CCK-8 assay and live/dead staining demonstrated no significant differences in cell viability, live cell ratio and cellular morphology (Supplementary Figures S12–S14), suggesting that moderate TMs had no toxicity to L929 cells. As TM volume fractions increased to over 10‰, the cell viability significantly decreased, which was possibly because microgels occupied excessive space for cellular growth and proliferation. Intriguingly, when the co-culture systems were previously illuminated for 30 min before further co-culture, transient proliferation promotions were observed, possibly due to cellular utilization of the TM products.

Conclusively, the concept of using TM to construct an artificial light-controlled energy supply module was demonstrated feasible. The module could produce ATP under light and supply energy for ATP-dependent processes both inside and outside it. Furthermore, the module exhibited low toxicity *in vitro*, indicating its *in vivo* application potential in the future.

However, it should be noted that the current study is primarily a proof-of-concept study under controlled *in vitro* conditions. Although the non-toxicity of the energy supply modules observed in cell cultures is promising, the performance and biocompatibility of the module have not been validated in more complex physiological environments, such as in animal models or *ex vivo* tissues. Factors including immune response, stability in biological fluids and functional efficacy *in vivo* remain to be thoroughly investigated. Furthermore, the feasibility of TMs powering more complicated reaction systems *in vivo* still requires following experiments to explore. These limitations highlight the need for further studies to better assess the practical potential and safety of the module for real-world biomedical applications.

## Conclusion and discussion

We report the successful encapsulation of thylakoids into Alg/Gel microgels to create artificial light-controlled energy supply modules. The encapsulation stabilized thylakoids via preserving their bioactivity and structural integrity, and TM showed light-responsive electron transfer of PSII and ATP production. Furthermore, TM can shield oxidative damage, impede the degradation of proteins and Chl and maintain structural integrity, thereby prolonging the activity retention of thylakoids. This preservative effect is correlated to the relatively high precursor concentrations, which resulted in the dense networks of microgels. Most importantly, the concept of artificial light-controlled energy supply modules based on microgels was validated as TM successfully triggered the luciferin/luciferase reaction both inside and outside the modules. Moreover, with excellent cytocompatibility, TM shows promising potential in application for therapies *in vivo*.

Among currently widely used hydrogels with excellent biocompatibility for supporting living system, such as gelatin methacryloyl, poly(ethylene glycol), alginate and their hybrids [54–56], Alg/Gel exhibits a particularly promising potential for constructing artificial photo-driven modules. Compared to biomaterials requiring light or chemical initiation for cross-linking, which may interfere with photoenzymatic components, Alg/Gel relies on calcium ions that have minimal impact on thylakoid activity at cross-linking concentrations [35]. Furthermore, owing to its temperature-adjustable viscosity, its precursor solution owns a low sol-gel transition point [57], thereby reducing the risk of thermal damage to structural integrity of thylakoid membrane and the activity of enzyme during gelation.

For long-term test (over a week), the freezing strategy at −80°C is currently the primary choice as it ensures the photosynthetic activity of thylakoids for at least half a year [23]. However, its application is hampered by two drawbacks. First, it relies on DMSO, a cryoprotectant, to prevent ice damage during freezing and thawing. Due to the non-negligible cytotoxicity of DMSO [58], multiple washing steps through centrifugation are indispensable when thawing, which may damage thylakoid viability. Second, thylakoids stored by this approach are not fully preserved (around 70% remain intact) [59], which means that a re-quantification is needed prior to each use. By comparison, the microgel encapsulation offers a ready-to-use strategy, while shielding thylakoids from potential external damages. Throughout the monitored lifespan of TM, almost no thylakoids were released and their Chl content remained stable, thereby eliminating the need for re-quantification.

Compared with other encapsulation strategies for constructing artificial energy modules, the microgel strategy not only integrates long-term activity and efficient energy output, but also demonstrates superior performance in activity preservation. For instance, coacervates formed via liquid–liquid phase separation led to a thylakoid activity loss of up to 30% within 1 day [16]. Although liposomes offer better protection than coacervates, their efficacy (75% activity retention after 4 days) is still lower than that of microgels (82% activity retention after 4 days) [17]. Given that artificial energy supply modules are often required to operate continuously in extracellular environments for extended periods, the microgel strategy, with superior operational simplicity and stability, presents a more suitable and robust approach for maintaining the activity of thylakoids or other sub-cellular structures in non-frozen conditions.

For *in vivo* application, a series of cross-kingdom challenges, particularly the immune reactions, still need to be addressed. While existing biomedical applications of chloroplast-derived agents did not show severe immune responses, the dosage-dependent behavior remains unclear [60–62]. Therefore, although over 97% of thylakoids were strictly confined within microgels, the few released ones might trigger negative effects. To mitigate any immune reactions, optimizing material ingredients (e.g. increasing cross-linking density or introducing other cross-linkers) to further enhance microgel stability and reduce thylakoid release is a possible solution. If necessary, encapsulating moderate immunosuppressants could also be considered [63]. In addition, Chen *et al.* have adopted a biomimetic strategy by encapsulating nanoscale thylakoid units into chondrocyte membranes for camouflage, providing an innovative method to prevent cross-kingdom issues [20]. With these potential solutions for immune reactions, the future application of thylakoid-based biomaterials is promising.

In this research, how to utilize the ATP for therapeutic purposes remains a challenge and requires further exploration. During the photoenzymatic processes on thylakoid membrane, three major products are generated, including O_2_, NADPH, and ATP, all of which play crucial roles in supporting cell life. Among them, O_2_ supplied by thylakoids or thylakoid-derived biomaterials has shown excellent effects in promoting wound healing by mitigating hypoxia [22, 60]. Compared to O_2_, NADPH, and ATP are more multi-functional as they deeply participate in various intercellular enzymatic processes [64, 65]. However, thylakoids may lose the essential co-factor for NADPH regeneration, ferredoxin, significantly reducing the NADPH production capacity after isolation [23]. This was validated by our results that showed the NADPH yield was only few dozen micromoles, which might be insufficient for application. While adding external ferredoxin could effectively restore NADPH yield, the cost was relatively high. Given that, searching for a substitution for ferredoxin to maintain the electron transfer would be more cost-effective. The biotic-abiotic hybrid strategy reported by Xiong *et al.* is encouraging. They integrated CdTe quantum dots into thylakoids as artificial electron carriers, significantly increasing NADPH yield by ∼10-fold [21]. However, the safety of CdTe quantum dots requires further investigation as they were introduced for carbon fixation rather than biomedical scenes. If the limited NADPH yield could be addressed through a biocompatible pathway, the application of thylakoid-based artificial living systems would be markedly expanded.

Regarding the utilization of external ATP, the intrinsic polarity of ATP molecules severely limits their transmembrane efficiency, necessitating carriers for cellular uptake [66, 67]. To directly utilize the ATP synthesized by thylakoids, Chen *et al.* developed an innovative strategy of incorporating thylakoids into rat cells as a metabolic modulator by shearing intact thylakoids into nano-scale vesicles and then modifying them with cellular membranes [20]. In our view, ATP, the universal biochemical energy currency, may be indirectly utilized by transferring its energy to other high energy chemicals. For instance, high-energy phosphate groups could be transferred from ATP to phosphocreatine, a membrane-permeable energy carrier, thus allowing the energy supply for cells from external energy modules [68]. Cell-free biosynthesis systems can be considered as well [69]. As the light-driven cell-free protein synthesis (CFPS) has been validated in liposome-based modules [18], the synthesis of some small bioactive molecules or even proteins via cell-free systems powered by the microgel-based energy supply modules shows great potential. One limitation of TMs for CFPS is the inadequate ATP level. Existing CFPS system typically contains millimolar levels of ATP to support transcription and translation [70], which is much higher than the ATP production level (hundreds of micromoles) shown in this work. To match the energy demands for CFPS synthesis, ATP-producing capacity of TMs may be directly increased by encapsulating more thylakoids (e.g. over 100 μg mL^−1^ Chl equivalent). Introducing additional electron carriers, such as quantum dots, to significantly enhance photoenzymatic efficiency is also an alternative strategy [21]. Apart from enhancing ATP production, another perspective is that TMs can function as an energy regeneration system rather than the sole energy supply origin. In conventional CFPS systems, 3-phosphoglyceric acid is typically added to maintain protein synthesis by regenerating ATP after initial ATP is exhausted [71]. Compared to 3-phosphoglyceric acid-based systems, TMs eliminate the reliance on substrate, offering a sustainable and environmentally friendly energy regeneration system. Our following research aims to make full use of the produced ATP, thus applying the artificial light-controlled energy supply modules in cells for therapeutic purposes.

Additionally, our research demonstrated a promising strategy to construct living materials by blurring the borders between biosynthesis and materials. Therapies based on living systems, including cell therapy and organelle transplantation, have achieved prominent success. However, facing the challenges including safety, heterogeneity, ethical issues, shortage of donors and high cost, the further application of natural living systems is restricted [44, 72]. With lower immunogenicity, higher controllability and biomimetic functions, artificially constructed living systems with smart biomaterials such as coacervates and liposomes have been considered as a substitution [1, 7, 9, 73]. Our study introduced an alternative with excellent biocompatible microgels, and revealed their special properties in this field: greatly extending the lifetime of artificial living systems and realizing the exchange of substances, while ensuring the compartmentalization—a critical feature for mimicking natural tissues and avoiding cross-contamination.

## Supplementary Material

rbaf106_Supplementary_Data
