# Supplementary material for: A microgel-stabilized, light-controlled artificial energy supply module for efficient biosynthesis
Source: Regen Biomater. 2025 Nov 8;13:rbaf106. doi: 10.1093/rb/rbaf106 (PMC12883868; doi:10.1093/rb/rbaf106)
Supplement: rbaf106_Supplementary_Data [file rbaf106_supplementary_data.pdf]

A Microgel-Stabilized, Light-Controlled Artificial Energy Supply Module for Efficient Biosynthesis

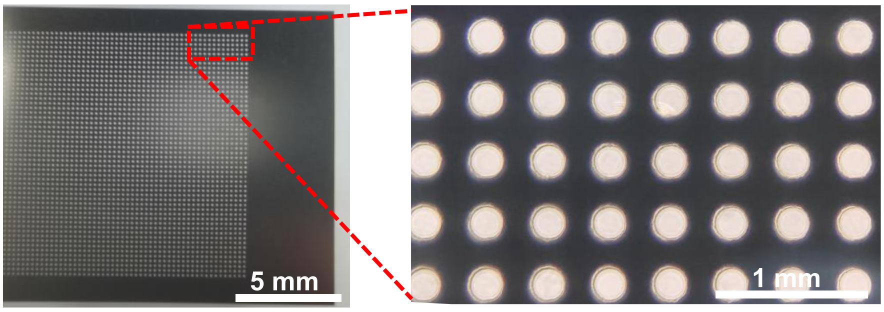

Figure S1. Morphology of the micromold. The micrmold was made by stainless steel and fabricated by lasing cutting to create a circular hole array, within which each hole was in equal size (200 μm × 200 μm)

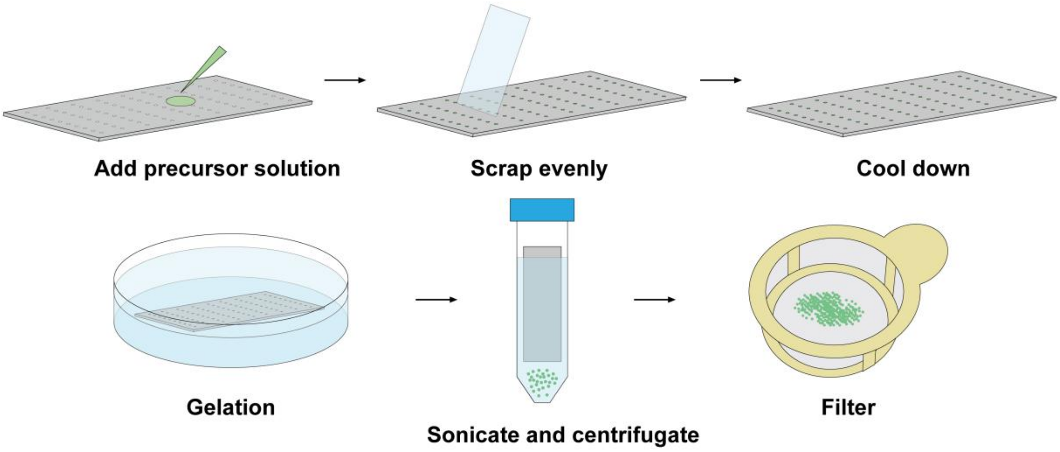

Figure S2. Preparation of microgels through micromolds. The microgels were shaped by the circular holes within the micromold, subsequently crosslinked through cooling down and immersing in calcium chloride solution, and finally collected using a cell strainer.

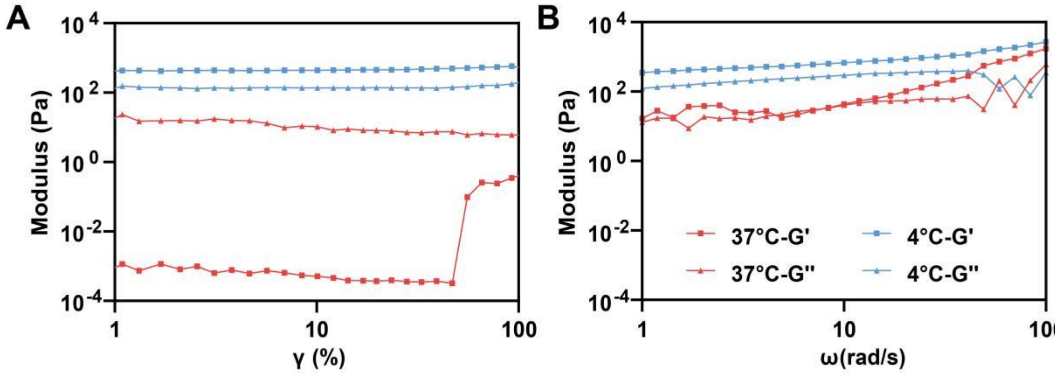

Figure S3. Rheological analysis of the precursor solution (4% Alg/Gel) at different temperatures: (A) strain sweep scanning from 1% to 100%; (B) angular frequency sweep scanning from 1 rad/s to 100 rad/s.

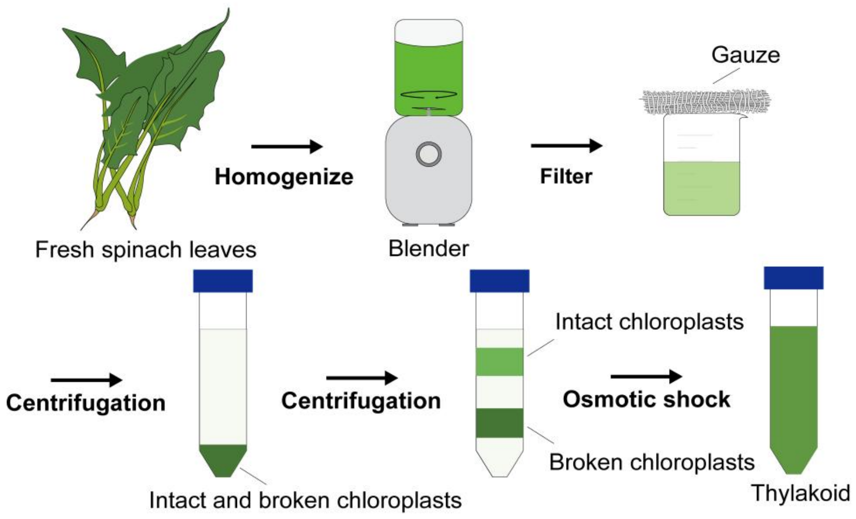

Figure S4. Extraction of thylakoid membranes. The thylakoid membranes were isolated from fresh spinach leaves purchased from the local market. Briefly, the leaves were homogenized, filtered, and centrifuged to gain thylakoid membrane suspension.

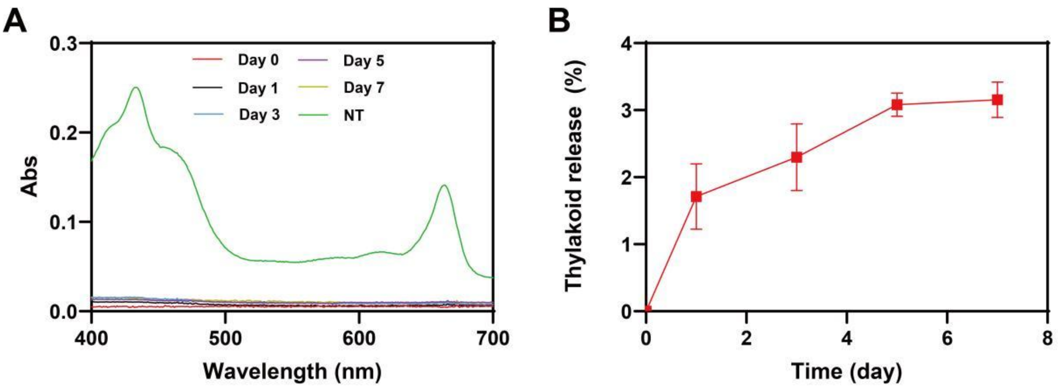

Figure S5. Thylakoid release kinetics from TMs: (A) UV-Vis absorption spectra of storage buffers from day 0 to day 7 compared to NT with equivalent Chl; (B) Quantification of the cumulative thylakoid release profile (n=3, mean ± s.d.).

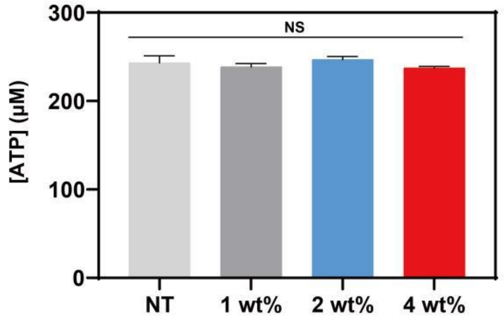

Figure S6. ATP production by NT and TMs with increasing precursor concentrations (15 μg mL^{-1} Chl equivalent) under white light (the light intensity was 25 W m^{-2}, n=5, mean ± s.d.). The ATP production of TMs was measured via quantifying ATP concentrations in the reaction buffers. NS (no significance): $P > 0.005$. P values were calculated by one-way analysis of variance (ANOVA).

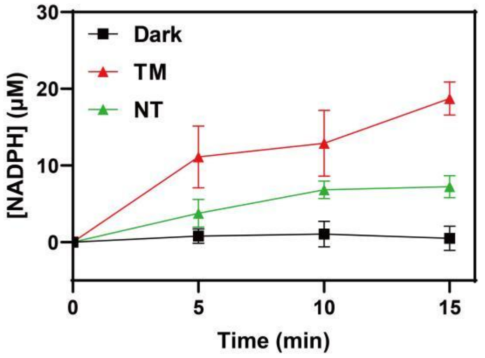

Figure S7. NADPH production by NT and TMs (15 μg mL^{-1} Chl equivalent) under white light (the light intensity was 25 W m^{-2}, n=3, mean ± s.d.) or in the dark.

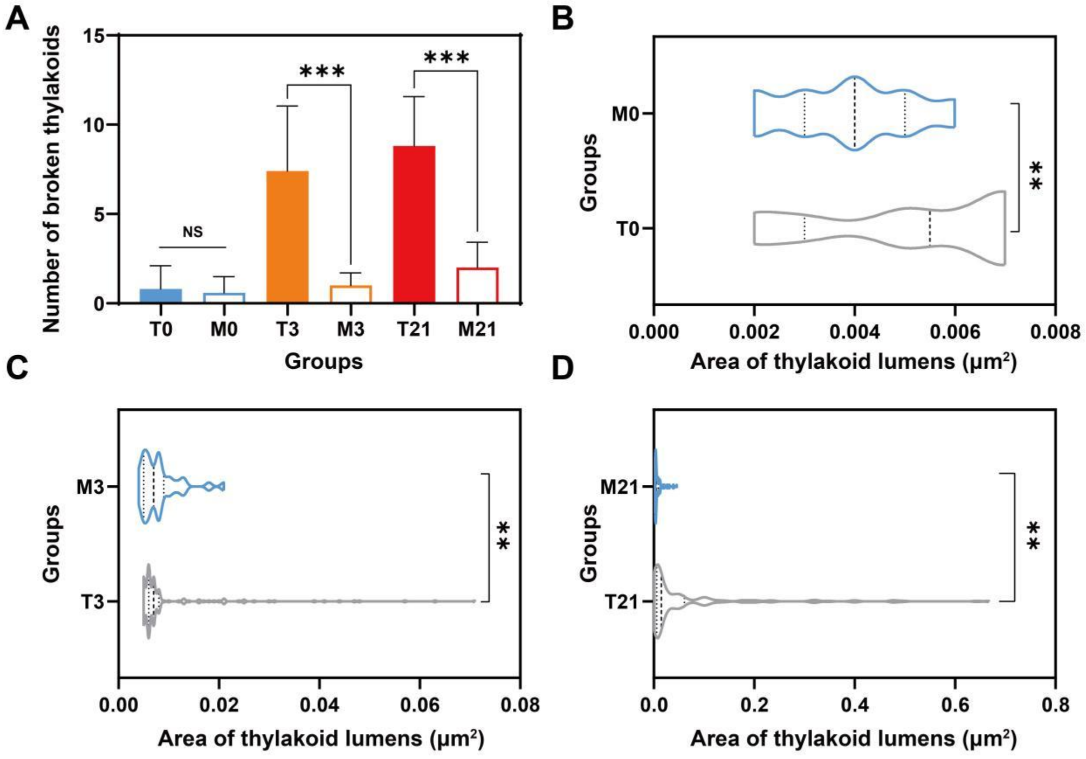

Figure S8. Quantity analysis of thylakoid membrane structure (A) average number of broken thylakoids per view under TEM (each view was a 5.8 μm × 5.8 μm square, n=5) and area distribution of thylakoid lumens in NT (T0, T3, and T 21) and TM (M0, M3, and M21) at (B) Day 0, (C) Day 3, and (D) Day 21. **: P < 0.01, ***: P < 0.001, NS (no significance): P > 0.005. P values were calculated by student's t test or

ANOVA.

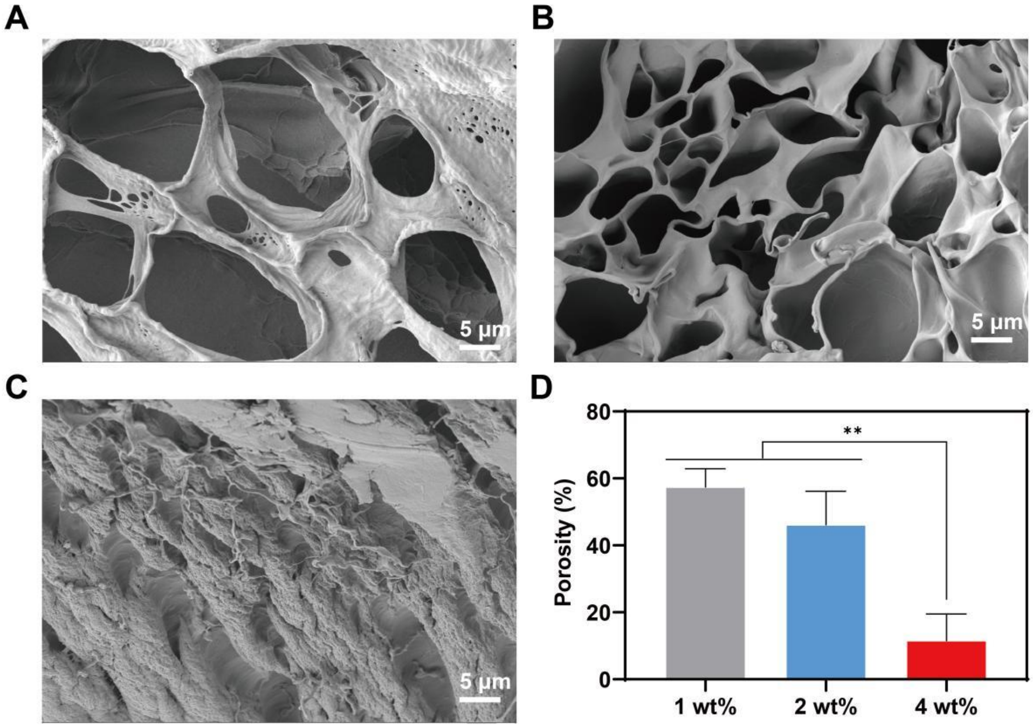

Figure S9. Representative morphology of TM with various precursor concentrations after freeze drying under scanning electron microscope: (A) 1 wt%, (B) 2 wt%, (C) 4 wt%, and (D) their porosity analysis results through ImageJ. The magnification times were all 2000 ×. **: P < 0.01. P values were calculated by ANOVA.

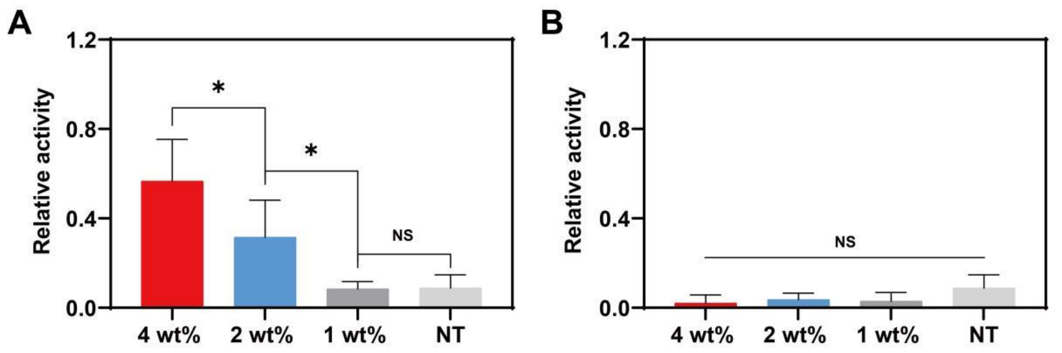

Figure S10. PSII activity retention of TM with different precursor concentrations and NT when incubated with 100 μM DCMU (A) distributed in the solution and (B) encapsulated within the microgels (Relative activity was defined as the ratio between the DPIP reducing capacity of the samples and that of freshly-isolated NT without DCMU; n=6, mean ± s.d.). *: $P < 0.05$, NS (no significance): $P > 0.005$. P values were calculated by ANOVA.

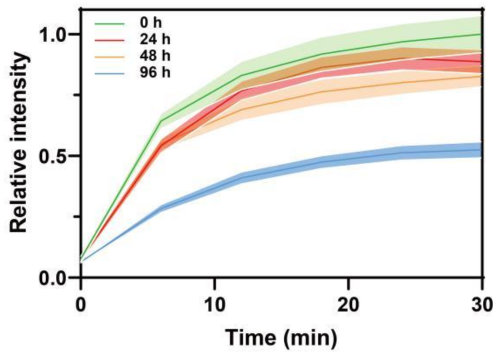

Figure S11. Changes of luciferin/luciferase reaction systems based on energy supply modules over time (the light intensity was 25 W m^{-2}; n=4, mean ± s.d.). The luminescence intensities were normalized to relative intensities.

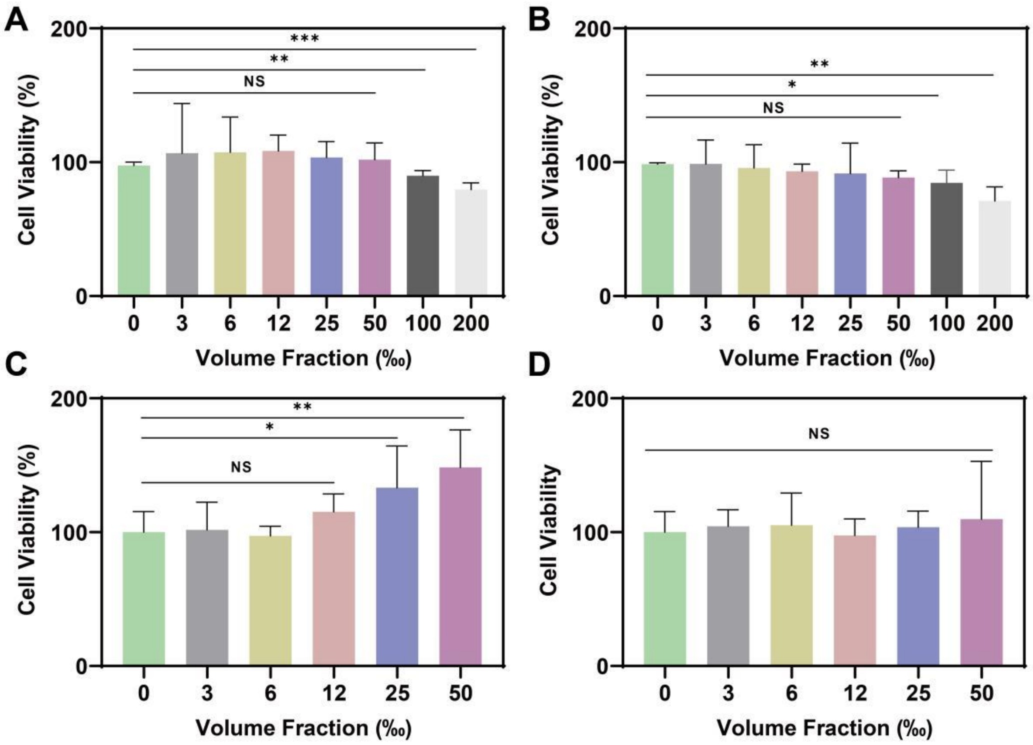

Figure S12. Viability of L929 cells co-incubated with different volume fractions of TMs (n=5, mean ± s.d.). (A) and (B): Cell viability after 24 hours and 48 hours of co-incubation in the dark; (C) and (D): Cell viability after 30 minutes illumination (the light intensity was 25 W m^{-2}) followed by 24 hours and 48 hours of further co-incubation in the dark. The cell viability was measured through CCK-8. NS (no significance): $P > 0.005$, *: $P < 0.05$, **: $P < 0.01$. P values were calculated by Student's t test.

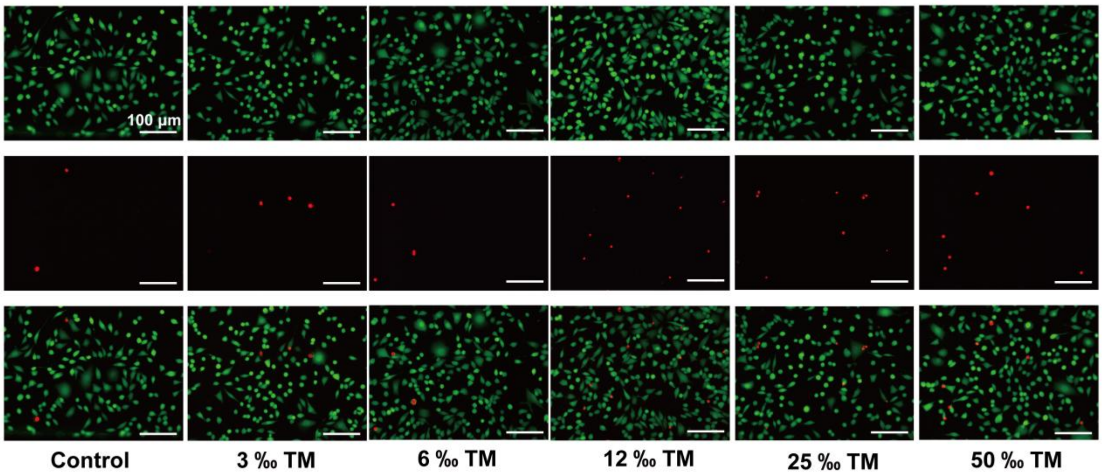

Figure S13. Live/Dead staining of L929 cells co-incubated with different volume fractions of TM.

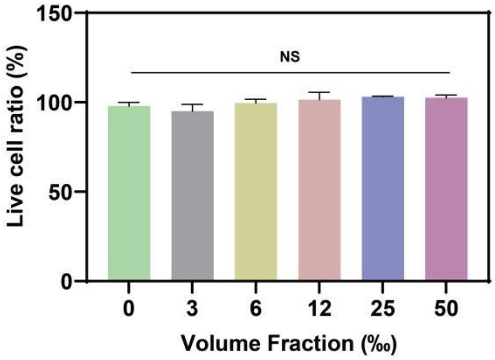

Figure S14. Live cell ratio (live cell number to the total cell number) of L929 cells co-incubated with different volume fractions of TM for 24 hours (n=5, mean ± s.d.). NS (no significance): $P > 0.005$. P values were calculated by ANOVA.
